# Nutritional Treatment of Hypertriglyceridemia in Childhood: From Healthy-Heart Counselling to Life-Saving Diet

**DOI:** 10.3390/nu15051088

**Published:** 2023-02-22

**Authors:** Maria Elena Capra, Giacomo Biasucci, Giuseppe Banderali, Cristina Pederiva

**Affiliations:** 1 Centre for Pediatric Dyslipidemias, Pediatrics and Neonatology Unit, University of Parma, Guglielmo da Saliceto Hospital, 29121 Piacenza, Italy; 2 Department of Translational Medical and Surgical Sciences, University of Parma, 43126 Parma, Italy; 3 Società Italiana di Nutrizione Pediatrica, 20126 Milan, Italy; 4 Department of Medicine and Surgery, University of Parma, 43126 Parma, Italy; 5 Clinical Service for Dyslipidemias, Study and Prevention of Atherosclerosis in Childhood, Pediatrics Unit, ASST-Santi Paolo e Carlo, 20142 Milan, Italy

**Keywords:** hypertriglyceridemia, nutrition, childhood, pediatrics, overweight

## Abstract

Hypertriglyceridemia is a lipid disorder with a varying prevalence; it is very common if we consider triglyceride plasma values slightly above the threshold, whereas it is extremely rare if only severely elevated triglyceride levels are considered. In most cases, severe forms of hypertriglyceridemia are caused by genetic mutations in the genes that regulate triglyceride metabolism, thus leading to extreme triglyceride plasma values and acute pancreatitis risk. Secondary forms of hypertriglyceridemia are usually less severe and are mainly associated with weight excess, but they can also be linked to liver, kidney, endocrinologic, or autoimmune diseases or to some class of drugs. Nutritional intervention is the milestone treatment for patients with hypertriglyceridemia and it has to be modulated on the underlying cause and on triglyceride plasma levels. In pediatric patients, nutritional intervention must be tailored according to specific age-related energy, growth and neurodevelopment requests. Nutritional intervention is extremely strict in case of severe hypertriglyceridemia, whereas it is similar to good healthy nutritional habits counselling for mild forms, mainly related to wrong habits and lifestyles, and to secondary causes. The aim of this narrative review is to define different nutritional intervention for various forms of hypertriglyceridemia in children and adolescents.

## 1. Introduction

Hypertriglyceridemia (HTG) is a common lipid disorder in childhood. Pediatricians and general practitioners are often very worried when they find moderately elevated triglyceride (TG) values in children and adolescents, and hypertriglyceridemia is a frequent reason for referral to a Lipid Clinic. In fact, severe hypertriglyceridemia raises some concerns in childhood and adolescence, as it represents a concrete threat of acute pancreatitis; moreover, patients with moderately altered values need to be taken care of in order to avoid consequences in the following years. In the adult population, HTG has been linked to an increase in atherosclerotic cardiovascular disease (ASCVD) [1,2,3]. Even if hypercholesterolemia needs to be addressed more promptly in childhood and adolescence in order to reduce cardiovascular disease risk, especially in subjects with familial hypercholesterolemia [4], HTG must not be underdiagnosed and undertreated either, in order to avoid cumulative cardiovascular disease risk later in life. Nutritional intervention is a milestone in the treatment of all forms of hypertriglyceridemia. Nutritional treatment must be tailored and personalized according to the age of the patient, the etiology of hypertriglyceridemia, and triglyceride plasma values. The most common cause of HTG in childhood is weight excess, yet in children with overweight or obesity, TG plasma levels are usually only slightly altered; therefore, in this category of patients, nutritional intervention is mainly aimed at achieving healthy-heart nutritional habits and lifestyle. Genetic forms of HTG are much less common, but in these cases, nutritional intervention must be tailored to each patient’s TG levels and clinical history, as it can be literally life-saving. The aim of our review is to analyze the main forms of hypertriglyceridemia in childhood and adolescence and to describe the nutritional intervention specific for each form.

## 2. Hypertriglyceridemia

### 2.1. Triglyceride Metabolism

**Figure 1:**
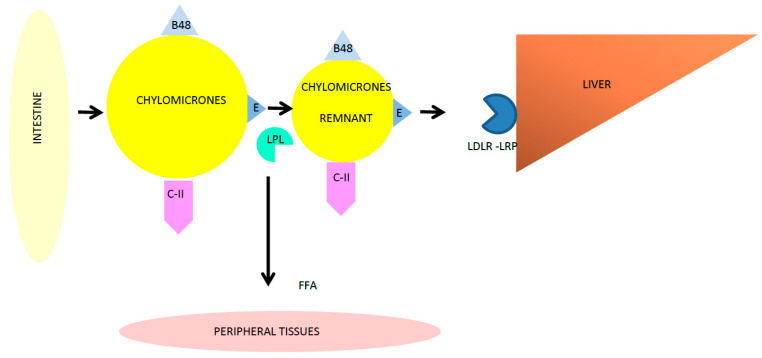
TG exogenous pathway.

TGs are present in lipoproteins containing Apolipoprotein B (ApoB), such as chylomicrons (CMs), CM remnants, and very-low-density lipoproteins (VLDL) [5]. Lipoproteins rich in TGs (TRLs, triglyceride-rich lipoproteins) bring TGs to peripheral tissues through two different pathways: the exogenous pathway (TGs derived from diet, summarized in Figure 1) and the endogenous pathway (TGs derived from the liver metabolism) [5].

In particular, TGs introduced through diet are absorbed in the small intestine; then, within one hour from the start of the meal, they are included in CMs and secreted into lymph. Apolipoprotein B48 (ApoB-48) is the main structural protein of CMs. CMs are hydrolyzed by lipoprotein lipase and become CM remnants. Lastly, the liver removes CM remnants from circulation through the bond of the low-density lipoprotein receptor (LDL-R) or LDL receptor-related protein (LRP). Thus, peripheral tissues can use free fatty acids (FFA) and glycerol, created during this process, as an energy source.

### 2.2. Classification of Hypertriglyceridemia

Hypertriglyceridemia is a form of dyslipidemia that can be defined as a high concentration of plasma TGs.

In 2010, the Endocrine Society elaborated on a more detailed classification, aimed at focusing attention on the possible incidence of HTG-related complications, such as pancreatitis [6] (Table 1).

In 2012, the European Society Consensus Panel adopted the following classification (reported in Table 2) [7].

The National Cholesterol Expert Panel (NCEP) ATP III classified TG levels in adults as listed in Table 3 [8].

## 3. Hypertriglyceridemia in Childhood and Adolescence

Acceptable and elevated plasma lipid levels in children and adolescents are reported in Table 4 [8]. TG plasma levels were evaluated according to patient’s age.

In the case of elevated TG levels, one of the classifications for the adult population reported in Table 1, Table 2 and Table 3 can be used to define the severity of HTG.

Elevated TG levels can be caused by a genetic mutation in the genes involved in TG metabolism (primary HTG) or by exogenous and environmental conditions that lead to an increase in TG circulating levels (secondary HTG) [9,10]. The main forms of primary and secondary HTG in childhood and adolescence are summarized in Table 5.

TG levels above 1000 mg/dL place patients at increased risk of acute pancreatitis, thus treatment must be started promptly. On the contrary, moderately elevated TG levels are often underestimated and undertreated [10,11], even if they are responsible in part for each individual’s cardiovascular risk. In the Bogalusa Study, high TG levels in childhood were associated with subsequent alteration of intima media thickness in adulthood [12]; this finding was also supported by data of the Pathological Determinants of Atherosclerosis in Youth study (PDAY) [13]. Therefore, HTG must always be considered as one of the determinants of cardiovascular disease risk in children and adolescents, and consequently treated according to its etiology and/or to TG plasma levels.

## 4. Nutritional Intervention in Pediatric Patients with Hypertriglyceridemia

Nutritional intervention is a milestone in the treatment of children and adolescents with hypertriglyceridemia. It is basically based on the restriction of lipid and simple sugars. The main longitudinal and intervention studies [14,15] conducted on this topic have confirmed that lipid restriction is safe at developmental age. However, in pediatric patients with severe HTG, lipid restriction is very strict; therefore, a close and tailored monitoring of growth and neurodevelopment is mandatory, especially in the first years of life. Lipid restriction should be partially compensated for by an increase in daily protein intake, because simple sugars must be reduced too. Nutritional counseling for pediatric patients with HTG is detailed, strict, and restrictive when TG plasma values are severely altered. On the contrary, nutritional intervention is similar to healthy life nutritional counseling when TG levels are only slightly altered, especially if there is a co-existence of overweight and/or bad nutritional habits. However, nutritional intervention must always be tailored to a patient’s age, taking into account each patient’s specific energy as well as macronutrient and micronutrient needs, so as to grant an adequate growth and neurodevelopment, as well as to respect each patient’s and family’s tastes and habits [16].

### 4.1. Macronutrients in the Treatment of HTG

As we have already mentioned, nutritional intervention for pediatric patients with HTG is aimed at modulating macronutrient intake, so as to reduce daily intake of lipid and simple sugars. Macronutrient intake should be modified as follows [17]:

- Fats: they are a concentrated energy source. Saturated fats and fatty acids have a negative influence on lipid profile, promoting an increase in atherogenic lipid fractions and in TG. They should be substituted with middle chain unsaturated fatty acids (MUFAs) and polyunsaturated fatty acids (PUFAs) [18].
- Carbohydrates: they represent the main nutritional energy source for healthy children. In nutritional treatment of patients with HTG, daily intake of carbohydrates must be slightly reduced, especially simple sugars, with a preferential intake of complex carbohydrates. In the case of severe HTG, simple sugars must be almost totally eliminated, whereas in intermediate forms, the total daily intake of simple sugars should not exceed 10% of total daily energy intake [17].
- Proteins: daily intake of foods rich in proteins and poor in fats (such as lean meats, egg white, poultry, and others) can grant an adequate protein intake, maintaining the prescribed lipid restriction. Some studies, conducted in adult patients, have highlighted that a hyperproteic diet is linked to relevant reduction in TG plasma levels. However, studies conducted in pediatric patients (CHOP) have showed an increase in overweight and obesity in patients with a high daily protein intake [19,20].

### 4.2. Main Food Categories and Dietary Patterns

Nutritional intervention in pediatric patients with HTG must always grant adequate energy intake, a balanced macronutrient intake, a correct distribution of nutrients in the three main daily meals and two snacks, and a good food variety [17,21]. In the clinical practice, it is advisable to have a regular daily intake of fruits and vegetables (limited only in the case of severe HTG), whole foods (pasta, rice, and other cereals), and pulses, with a moderate intake of lean meats and low-fat dairy products. Industrial products rich in saturated fatty acids and simple sugars, such as sweetened beverages and alcohol, should be banned. In the last few years, there has been growing evidence of the importance of considering foods not only as a sum of single nutrients, but rather as a part of a food matrix, shifting the focus from single nutrients to whole foods [22]. Nowadays, the so-called dietary pattern represents the most updated approach to dietary nutritional intervention, hence the importance of different dietary models, both historical and modern ones [23]. A Mediterranean Diet certainly represents a valid model for patients with HTG too, as it recommends a regular intake of fruits and vegetables, low-fat dairy products, lean meats, pulses, and olive oil. Other nutritional models have been considered as well, such as the Nordic Diet, the DASH Diet, and the vegetarian diet, which are particularly useful in order to respect different countries’ habits and heritage [17].

## 5. Nutritional Intervention in Primary and Secondary Forms of Hypertriglyceridemia

In this section, we will analyze nutritional intervention and its specific modulation in different forms of HTG.

### 5.1. Familial Chylomicronemia Syndrome

**Figure 2:**
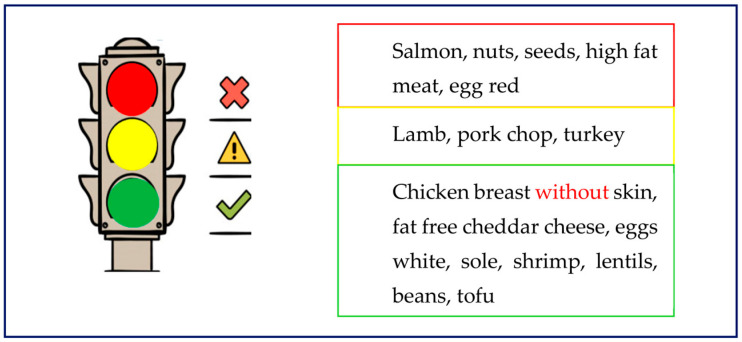
Protein-rich foods advised or not for patients with FCS, derived and adapted from [28].

Familial chylomicronemia syndrome (FCS) is a rare autosomal recessive monogenic disorder, affecting approximately two individuals in a million, caused by a mutation in the genes involved in the lipolytic cascade [24]. In four out of five patients with FCS, the mutation is on the gene encoding for lipoprotein lipase (LPL). In the other cases, the mutation is on the genes encoding for the co-factors or maturation proteins of LPL, namely lipase maturation factor 1 (LMF1), glycosylphosphatidylinositol-anchored high-density lipoprotein-binding protein 1 (GPIHBP1), apolipoprotein C2 (ApoC2), and apolipoprotein A5 (ApoA5) [25]. From a biochemical point of view, the abnormal persistence of circulating CM after at least a twelve-hour fasting period represents a distinct feature of FCS, which leads to lactescent fasting blood and fasting plasma TG levels above 1500 mg/dL, classified as severe HTG. Clinical presentation of FCS includes eruptive xanthomas, lipemia retinalis, splenomegaly, hepatomegaly, and acute or recurrent episodes of abdominal pain or pancreatitis [25]. FCS symptoms usually start in childhood, with pancreatitis as the most severe clinical complication, occurring in 50 to 80% of patients [7,26]. Nutritional intervention is a cornerstone in the treatment of patients with FCS. The aim of nutritional intervention is to keep TG levels below the threshold of 1000 mg/dL, above which CM concentration increases along with the risk of pancreatitis [27,28]. Patients with FCS are advised to adopt a very-low-fat diet with a total dietary fat intake lower than 15% of total daily energy intake. Fat restrictions must be individualized, especially in childhood, in order to meet the daily requirement of essential fatty acids (2 to 4% of daily energy intake). Fat intake should be distributed throughout all meals, so as to maintain TG levels as stable as possible [28]. The intake of low-fat or fat-free protein foods, such as egg white, shrimps, sole, beans, lentils, and breast poultry, should be encouraged, as it enables patients to reach adequate energy and macronutrients goals. Foods rich in unsaturated fats, such as salmon and nuts, are not recommended, as long-chain saturated fats, transfats, and unsaturated fats are metabolized using the LPL-dependent pathway [28]. Protein-rich foods that can or cannot be consumed by patients with FCS are summarized in Figure 2.

In childhood, growth and neurodevelopment must be strictly monitored in patients with FCS following a very-low-fat diet. Essential fatty acid adequate intake must be granted in order to avoid neurodevelopmental impairment and fat-soluble vitamin supplements are often necessary. A daily dietary plan is provided to the child and the family, with precise quantities of each food and possible substitutions, according to age, sex, and weight. General recommendations, subdivided by age, are summarized in Table 6 [29].

### 5.2. Multifactorial Chylomicronemia Syndrome

Multifactorial chylomicronemia syndrome (MCS) is defined as a condition characterized by the co-existence of genetic and secondary forms of severe HTG, with an estimated prevalence in the general population of 1 in 600 to 1000 individuals. MCS is a polygenic disorder exacerbated by external factors [30]. FCS and MCS are not always easy to distinguish from a clinical point of view, setting a real challenge for clinicians. Patients with MCS usually have a good response to lifestyle modification, to the treatment of secondary coexisting causes of HTG, and to TG-lowering drugs. On the contrary, patients with FCS do not show a significant improvement with the use of TG-lowering drugs, thus the nutritional treatment of these patients is much stricter [31]. The nutritional treatment of patients with MCS is similar to that of patients with FCS, and it should be adjusted according to TG levels.

### 5.3. Familial Combined Hyperlipidemia

Familial combined hyperlipidemia (FCHL) is one of the most common forms of genetic dyslipidemia; approximately 0.5 to 1% of the general population is affected by FCHL [32]. The genetic mutations underlying FCHL are still unknown and its phenotypic presentation results from the combination of genetic and environmental factors. Patients with FCHL have an increase in very-low-density lipoproteins (VLDL) and apolipoprotein B100 (ApoB-100) hepatic production and a decrease in CM remnants’ clearance. The blood lipid profile is variable but, usually, patients with FCHL have moderately elevated TG, normal or slightly elevated LDL cholesterol, and low HDL cholesterol levels. Family history of patients with FCHL is usually positive for dyslipidemia and/or premature coronary heart diseases [9]. Nutritional intervention in children and adolescents with FCHL is aimed at the optimization of body weight, while decreasing the intake of saturated and trans-fats. The lipid amount should be 25 to 30% of total daily energy intake, according to Italian Pediatric Nutrition Society (SINUPE) indications [33]. A healthy lifestyle, including adequate daily physical activity as well as avoidance of smoking and alcohol use, should be encouraged too.

### 5.4. Dysbetalipoproteinemia

Dysbetalipoproteinemia is a rare genetic disease caused by a homozygous mutation in the gene encoding for apolipoprotein E (ApoE). ApoE is a ligand for CM, for intermediate density lipoproteins (IDLs), and for VLDL hepatic receptors. When ApoE is defective, remnant particles (including CM) accumulate in the blood vessels, causing an increase in total cholesterol and in TG plasma levels. The clinical onset of dysbetalipoproteinemia usually occurs in young adulthood, but the co-existence of overweight, obesity, and/or diabetes can foreshadow it in adolescence or late childhood. Patients usually present with xanthomas on palmar crease or on pressure sites. Nutritional treatment is aimed at lowering TG levels, tailored to TG levels, and similar to that of patients with FCHL [9].

### 5.5. Weight-Excess-Related Hypertriglyceridemia

TG levels can be elevated not only on a genetic basis, but also as a secondary effect of other diseases. The main causes of secondary HTG are weight excess, liver or kidney diseases, or the use of specific medications [10]. In the last decades, overweight and obesity in childhood and adolescents have epidemically increased, posing a serious public health concern [34]. HTG has a higher prevalence in children and adolescents with overweight or obesity, and it can be classified as the most common dyslipidemia in these clusters of subjects [35]. HTG in pediatric subjects with weight excess has a multifactorial etiology: it derives both from TG adipocyte mobilization induced by insulin resistance action and from insulin-stimulated liver TG synthesis [10]. Non-alcoholic fatty liver disease (NAFLD) is a complication of weight excess that can already be detected in childhood. NAFLD is defined as excessive lipid accumulation in the liver in subjects with no co-existing liver disease and without reported excessive alcohol intake (less than 30 g per day in men and less than 20 g per day in women) [36]. This definition has been recently updated for adult subjects, switching from the definition of NAFLD to that of metabolic dysfunction-associated fatty liver disease (MAFLD) [37]. An international panel of experts has recently proposed diagnostic criteria for MALFD in pediatric subjects, but a consensus has not yet been reached [38]. The presence of obesity associated with HTG in childhood is related to an increase in both fatal and non-fatal cardiovascular events in adulthood [39]; therefore, an adequate intervention must be started as soon as possible. If TG levels are higher than 500 mg/dL, lipidology counseling is needed, otherwise the nutritional intervention is the same as that proposed to overweight and obese children. In children and adolescents with weight excess and HTG, nutritional intervention is mainly aimed at the achievement of optimal weight through the acquisition of age- and sex-adjusted correct nutritional and lifestyle behaviors [39].

In adult subjects with obesity, hypocaloric diets are related to a reduction in BMI and in TG levels [40]. In pediatric patients, hypocaloric diets are not routinely prescribed in the case of overweight or obesity. The first step of the evaluation should always be the assessment of the child’s and his or her family’s nutritional habits, tastes, and cultural background, in order to tailor the nutritional intervention as much as possible. Filling in a food diary is a good tool for assessing nutritional habits.

The main nutritional recommendations are summarized in Table 7 [34,39].

In pediatric subjects with weight excess and HTG, special attention should be paid to specific food items, as shown in Table 8.

According to recent FAO recommendations [23], to improve the lipid profile in children with overweight or obesity, nutritional intervention should consider foods not only as single nutrients, but as a part of a food matrix and of a dietary pattern.

## 6. Focus on Specific Nutrients

Some foods or food complements deserve special attention in the nutritional treatment of HTG, as sometimes they can be used to improve or to balance the diet; nevertheless, sometimes their intake must be limited. We focused our attention on omega-3 long chain polyunsaturated fatty acids (LCPUFAs) and on middle chain triglycerides for the lipid component, and on stevia and fructose with regard to the carbohydrate component [45,46]. According to food-based recommendations, their specific nutrients should be considered as a constituent part of the food matrix.

### 6.1. Long Chain Polyunsaturated Fatty Acids

LCPUFAs are fatty acids with 18 or more atoms of carbon; they are subdivided into two main families depending on the first double bond position from the methyl end group: (*n*-6) and (*n*-3). The main (*n*-3) LCPUFAs are α-linolenic acid (ALA), eicosapentaenoic acid (EPA), and docosahexaenoic acid (DHA), whereas the main (*n*-6) LCPUFAs are linoleic acid (LA) and arachidonic acid (ARA). DHA and EPA are the most widely studied LCPUFAs in cardiovascular prevention. DHA and EPA derive from ALA, which is an essential LCPUFA. They are considered as “conditionally essential”, as they can be synthesized from ALA, but, owing to a poorly efficient biochemical conversion, they also need to be taken from exogenous sources, that is, foods or supplements, in order to reach an adequate plasma level. DHA and EPA are converted into cell membrane phospholipids, and they are mostly present in the heart and brain [47]. The main dietary sources of DHA and their content in some foods are reported in Table 9 [48].

In the adult population with HTG, the American Heart Association recommends daily supplementation of 2 to 4 grams of EPA + DHA [1,2,48]. In a recent review [17], the authors investigated the effect of LCPUFAs on HTG in adult subjects. With respect to EPA, DHA seems to have a higher anti-inflammatory effect and a greater effect on insulin sensitivity, as it can upregulate genes involved in insulin sensitivity, glucose metabolism, and intracellular signaling [49,50]. In a randomized controlled trial conducted on children with NAFLD, DHA supplementation improved liver steatosis and insulin sensitivity, related to a reduction in plasma triglycerides [51]. In pediatric patients with obesity, the role of (*n*-3) LCPUFAs is still debated, and further studies are needed to assess their effect on metabolic patterns and to verify whether their anti-inflammatory and metabolic properties are also exerted in pediatric patients, as well as in the adult population [47].

### 6.2. Medium-Chain Triglycerides

Medium-chain triglycerides (MCTs) contain fatty acids with 8 to 12 carbon acids; they are directly absorbed into the portal circulation, bypassing the liver, thus sparing CM formation. MCTs are hydrolized in the gut mucosa to become MCT fatty acids; these in turn are bound by albumin and travel through the portal vein to the liver, where they are oxidized to ketones [52]. On the contrary, long chain fatty acids are absorbed from the gut and incorporated into CM, then entering the lymphatic stream. Therefore, MCTs are often used in the nutritional treatment of patients with severe HTG to improve the macronutrient balance and to provide additional energy intake. MCT should be introduced gradually, so as to avoid diarrhea or abdominal pain. Other conditions that counterindicate the use of MCT are those characterized by a low plasma albumin concentration, such as nephrotic syndrome or liver cirrhosis, and those diseases that can worsen with an increase in ketonic bodies, such as diabetes mellitus [53]. MCT oil is a medical food that is available with medical prescription; it should not be confused with other oils such as coconut oil, not suitable for patients with FCS as it contains lauric acid, which is metabolized through the CM pathway [27].

### 6.3. Fructose

In the general population, fructose intake mainly derives from caloric sweeteners, such as sucrose, honey, and apple juice, and only secondarily from fruits, vegetables, and nuts [54]. Fructose enters the blood stream more slowly than glucose, thus reaching lower plasma levels, but with a higher persistence. In the liver, it is converted to trioses, then it is released as lactate into the blood stream or enters the gluconeogenesis pathway to become glucose and then glycogen [55]. Fructose acts as a non-regulated substrate for liver de novo lipogenesis, thus contributing to a greater TG and VLDL production, when compared with the same amount of glucose in healthy adults [56,57]. Studies conducted in the adult population [58,59] showed a correlation between non-fasting triglyceride levels and cardiovascular risk. Non-fasting triglyceride levels seem to be a better predictor of cardiovascular risk than fasting triglycerides levels, thus focus has been placed on dietary factors that can contribute to the increase in post-prandial TG levels. In a recent meta-analysis [60], the role of fructose in TG metabolism has been analyzed. In isocaloric diets, fructose does not alter postprandial TG. On the contrary, in trials with hypercaloric diets, high consumption of fructose (i.e., fructose intake providing 25% energy excess with respect to background diet alone) is linked to an increase in post-prandial TG levels [61]. A study conducted in overweight schoolchildren has showed a link between higher fructose intake and smaller LDL particle size concentration, which is linked to a higher risk of atherogenic process [61]. In childhood, excessive consumption of sweetened and carbonated beverages has been linked to an increase in obesity, to insulin resistance, and to hyperinsulinism [36,62], and overconsumption of fructose has been linked to an increase in small and dense LDL [63]. Moreover, fructose has an epigenetic effect, as it can alter lipid metabolism gene expression, with an increased risk of NAFLD [64]. Indeed, increased consumption of sweetened beverages has been linked to the development and progression of NAFLD [65].

### 6.4. Stevia

Stevia rebaudiana is a sweet herb; its leaves have been used for centuries by indigenous Southern American populations to sweeten food and beverages. Steviol glycosides are four-ring diterpenes composed of a backbone called steviol, to which different amounts and kinds of sugars are attached. Steviol glycosides remain undigested throughout the stomach and the small intestine, then enter the colon to be hydrolized by the gut microbiota. The remaining steviol is absorbed and transported to the liver, where it is either conjugated to glucuronic acid to form steviol glucuronide or excreted in the feces. Highly purified steviol glycoside extracts have been approved in many countries as sweeteners for food industry products [66]. The ingestion of steviol glycosides has been linked to a reduction in plasma glucose levels and to an improvement in glucose tolerance in healthy adults [67]. The effect of steviol glycosides on lipid profile is still debated. Most of the existing literature indicates that 200 to 1500 mg/day of steviol glycoside intake does not have a statistically significant effect on lipid profile in healthy adults [68,69]. In one study conducted on adult patients with type 2 diabetes mellitus, the consumption of 1000 mg of steviol glycosides daily for two months resulted in a reduction in total cholesterol, triglycerides, and VLDL [70]. In a recent double-blind open label study conducted on normal weight and slightly overweight adult subjects, the consumption of a sugar blend (a combination of sugar and stevia) resulted in a decrease in blood TG with respect to the intake of common sucrose [71]. In a recent systematic review, a non-significant effect of steviol glycoside consumption on body mass index reduction, fasting blood glucose, and total cholesterol reduction was confirmed in non-diabetic adult subjects [72]. Unfortunately, no significant studies are available for children and adolescents. Up to now, there is no sufficient evidence to recommend the use of products containing steviol glycosides for pediatric patients with HTG.

## 7. Discussion and Conclusions

HTG is a rather frequent condition in clinical practice, as it has an overall prevalence of 10% in both the pediatric and adult populations [3]. Primitive genetic forms of HTG are rare, whereas secondary forms of HTG, owing to unhealthy lifestyles, overweight, or obesity, are becoming more and more frequent in the pediatric population. Nutritional intervention is a milestone in the treatment of patients with HTG; it always guarantees a reduction in TG plasma levels, and even their normalization in some cases. As reported in our paper, nutritional intervention for HTG has a wide range of actions, from a strict tailored diet for severe forms to nutritional counseling for mild forms secondary to overweight or obesity. We would advise pediatricians to always start a patient-tailored nutritional intervention, respecting each age’s nutritional needs as well as the patient’s and his/her family’s tastes, heritage, and habits. The feasibility of the proposed nutritional intervention in each specific family context must always be taken into great consideration. In the near future, we hope to further improve and personalize nutritional therapy for pediatric patients with HTG, thanks to nutrigenetic and nutrigenomic studies, allowing a precision and tailored nutrition starting from the first years of life [17,73,74].

## Figures and Tables

**Table 1:** Classification of TG plasma levels according to the Endocrine Society [6].

The Endocrine Society	
Normal TG	<150 mg/dL
Mild HTG	150–199 mg/dL
Moderate HTG	200–999 mg/dL
Severe HTG	1000–1999 mg/dL
Very severe HTG	≥2000 mg/dL

**Table 2:** Classification of TG plasma levels according to the European Society Consensus Panel [7].

The European Society Consensus Panel	
Normal	<150 mg/dL
HTG	150–885 mg/dL
Severe HTG	≥885 mg/dL

**Table 3:** Classification of TG plasma levels according to the National Cholesterol Expert Panel [8].

NCEP ATP III	
Normal TG	<150 mg/dL
Borderline-high TG	150–199 mg/dL
High TG	200–499 mg/dL
Very high TG	≥500 mg/dL

**Table 4:** Acceptable and high plasma lipid values in children and adolescents [8].

Test	Acceptable	Borderline	High
Total cholesterol	<170	170–199	≥200
LDL-cholesterol	<110	110–129	≥130
Triglycerides			
0–9 years	<75	75–99	≥100
10–19 years	<90	90–129	≥130
HDL-cholesterol	>45	40–45	<40

**Table 5:** Primary and secondary forms of HTG in childhood and adolescence [9,10].

Forms of HTG	
Primary	Secondary
Familial Chylomicronemia Syndrome Multifactorial Chylomicronemia Syndrome Familial Combined Hyperlipidemia Dysbetalipoproteinemia	Overweight/obesity Maternal/gestational factors Drugs or medications Type 1 or type 2 diabetes mellitus Liver, kidney, endocrine, and immune disorders

**Table 6:** Dietary recommendations for pediatric patients with FCS (derived and adapted from [29]).

Age	Recommendation
Infant (0–12 months)	Skimmed and fortified expressed breast milk or special low-fat, low-LCT, high-MCT formula milk Start complementary feeding with low-fat food Supplementation of EFA and fat-soluble vitamins
Toddler (12–24 months)	Transition from breast milk or formula milk to skimmed cow milk Age-appropriate, low-fat solid food. Prefer vegetables, lean meats, complex carbohydrates. MCT oil
School-age children and adolescents	Recommendations adjusted for age, growth, and caloric goals Use MCT oil Medical letters may be written to school to modify food provided as meals and snacks

**Table 7:** Dietary recommendations for pediatric patients with overweight or obesity, adapted from [34,39].

Dietary Recommendations for Pediatric Patients with Weight Excess	
Item	Specific Recommendation
Number of meals	3 main meals and 2 snacks per day, breakfast every day
Type of carbohydrates	Substitute simple sugars with complex carbohydrtaes,
Type of snacks	Avoid high-energy and low-nutrient food, no sugar-sweetened beverages
Fruits/vegetables	5 portions per day
Energy and macronutrients intake	Daily energy and macronutrient intake should fulfill the National Recommended Energy and Nutrient Intake Levels according to gender, age, and ideal weight for stature: protein 1 g/kg/day, carbohydrates 45–60% of total daily energy, lipids 20–30% of total daily energy, saturated fatty acids <10% of total daily energy

**Table 8:** Specific food items for subjects with weight excess.

Food Item	Study	References
Sugar sweetened beverages	- Cross-sectional study on 2032 children (age 7–18 years): sugar-sweetened beverage consumption is linked to a higher risk of obesity and HTG in children	He et al. 2018 [41]
	- Meta-analysis of 33 studies: sugar-sweetened beverage consumption is linked to an increase in BMI, waist circumference, and body fat percentage in children	Farhangi et al. 2022 [42]
Saturated fats	- U.S. guidelines: reduction in saturated fat intake to less than 10% of total daily energy intake	U.S. guidelines 2020 [43] WHO guidelines [44]
	- Consider not only single nutrients, but also the food matrix in which saturated fats are inserted	FAO guidelines 2021 [23]

**Table 9:** Docosahexaenoic acid (DHA) content of some fish/seafood and animal meat [48].

Lipid and DHA Content in Fish, Seafood, and Meat		
Food	Lipids (g) per 100 g of Food	DHA % of Total Lipids
Cod, deep frozen, roasted in oven	0.9	38.65
Round Sardinella, fresh	4.5	29.29
Tuna, fresh	8.1	26.54
Sole, fresh	1.4	25.97
Salmon, fresh	12	11.27
Liver, chicken, raw	6.3	4.78
Turkey, whole, with skin, raw	6.9	4.54
Beef, front part cuts	7	1.50
Beef, flank steak and brisket, lean only	10.2	1.48
Turkey, leg, with skin, raw	6	1.48
Beef, sirloin-steak, lean only	5.2	1.34
Beef, rib, lean only	6.1	1.33
Chicken, whole, with skin, raw	10.6	0.72

## Data Availability

Not applicable.

## Abbreviations

ALA	Alfa linoleic acid
ApoA5	Apolipoprotein A5
ApoB	Apolipoprotein B
ApoC2	Apolipoprotein C2
ApoE	Apolipoprotein E
ARA	Arachidonic acid
ASCVD	Atherosclerotic cardiovascular disease
BMI	Body mass index
CM	Chylomicrons
DHA	Docosaexahenoic acid
EPA	Eicosapentaenoic acid
FAO	Food and agricultural organization
FCHL	Familial combined hyperlipidemia
FCS	Familial chylomicronemia syndrome
FFA	Free fatty acid
GPIHBP1	Glycosylphosphatidylinositol-anchored high-density lipoprotein-binding protein 1
HDL	High density lipoprotein
HTG	Hypertriglyceridemia
IDL	Intermediate density lipoprotein
LA	Linoleic acid
LCPUFA	Long chain polyunsaturated fatty acid
LDL	Low density lipoprotein
LDL-R	Low density lipoprotein receptor
LMF1	Lipase maturation factor 1
LPL	Lipoprotein lipase
MAFLD	Metabolic dysfunction associated fatty liver disease
MCS	Multifactorial chylomicronemia syndrome
MCT	Medium chain triglyceride
NAFLD	Non-alcoholic fatty liver disease
NCEP	National Cholesterol Expert Panel
PDAY	Pathological Determinants of Atherosclerosis in Youth
SINUPE	Società Italiana di Nutrizione Pediatrica
TG	Triglyceride
WHO	World Health Organization
